# Genetics of Type 2 Diabetes: Insights into the Pathogenesis and Its Clinical Application

**DOI:** 10.1155/2014/926713

**Published:** 2014-04-17

**Authors:** Xue Sun, Weihui Yu, Cheng Hu

**Affiliations:** ^1^Shanghai Diabetes Institute, Shanghai Clinical Center for Diabetes, Shanghai Key Clinical Center for Metabolic Disease, Shanghai Key Laboratory of Diabetes Mellitus, Shanghai Jiao Tong University Affiliated Sixth People's Hospital, Shanghai 200233, China; ^2^Department of Endocrinology and Metabolism, Wenzhou Medical University Affiliated First Hospital, Wenzhou 325000, China; ^3^Shanghai Jiao Tong University Affiliated Sixth People's Hospital, South Branch, Shanghai 200233, China

## Abstract

With rapidly increasing prevalence, diabetes has become one of the major causes of mortality worldwide. According to the latest studies, genetic information makes substantial contributions towards the prediction of diabetes risk and individualized antidiabetic treatment. To date, approximately 70 susceptibility genes have been identified as being associated with type 2 diabetes (T2D) at a genome-wide significant level (*P* < 5 × 10^−8^). However, all the genetic loci identified so far account for only about 10% of the overall heritability of T2D. In addition, how these novel susceptibility loci correlate with the pathophysiology of the disease remains largely unknown. This review covers the major genetic studies on the risk of T2D based on ethnicity and briefly discusses the potential mechanisms and clinical utility of the genetic information underlying T2D.

## 1. Introduction

The prevalence of type 2 diabetes (T2D) is rising rapidly owing to increased economic growth and lifestyle changes in both developed and developing countries. According to a recent report, the number of diabetics is estimated to reach 439 million by 2030 worldwide [1]. Therefore, strategies to prevent and treat diabetes are urgently needed in order to stem this global pandemic. It is well known that T2D is caused by *β*-cell dysfunction and/or insulin resistance, which is promoted by multifactorial genetic or environmental factors. Over the years, linkage analysis, candidate gene approach, large-scale association studies, and genome-wide association studies (GWAS) have successfully identified multiple genes that contribute to T2D susceptibility. Combined analyses of these loci, such as construction of genetic risk scores, have contributed significantly to the prediction of T2D diabetes and thus facilitated the adoption of early diagnosis and preventative strategies to reduce this growing disease burden [2–5].

Pharmacogenomics is an emerging discipline that highlights the role of inherited and acquired genetic variations in drug response and which is beneficial for appropriate selection of antidiabetic drugs [6]. So far, pharmacogenomics has proven to be valuable in guiding therapeutic choices in maturity onset diabetes in the young (MODY) and in neonatal diabetes; however, its extension to T2D still needs detailed studies [7]. The present review summarizes recent genetic research on T2D in both ethnic and chronologic contexts and briefly discusses the potential mechanisms and clinical utilities of genetic information in T2D.

## 2. Advances in Type 2 Diabetes Genetic Research 

Linkage analysis, candidate gene approach, large-scale association studies, and GWAS have identified approximately 70 loci conferring susceptibility to T2D. Among them, 45 loci were identified in European populations (Table 1), and the other 29 loci were identified in Asian populations, especially in East and South Asians (Tables 2 and 3). The immediate benefit derived from these findings was the better understanding of the pathophysiology of T2D.

### 2.1. Genetics of Type 2 Diabetes in European Populations

#### 2.1.1. Linkage Analysis, Candidate Gene Approach, and Large-Scale Association Studies

Linkage analysis has proved to be valuable in the exploration of genetic factors of monogenic diseases, such as MODY, neonatal mitochondrial diabetes, insulin resistance, and Wolfram syndromes [38–40]. However, it has not been particularly useful in identifying the genetic factors for common forms of T2D. Over the years, linkage studies have reported many predisposing associations with chromosomal regions for T2D, including segments in chromosomes 5 and 10, and have identified putative, causative genetic variants in* CAPN10 *[41],* ENPP1 *[42],* HNF4A *[43, 44], and* ACDC* (also called* ADIPOQ*) [45], but most of the findings from these reports could not be replicated.

During the past several decades, only a few loci conferring risk of T2D were identified through candidate gene approach with* PPAR*γ** Pro12Ala polymorphism being the first reported locus [8]. PPAR*γ* is a transcription factor that plays a pivotal role in adipocyte differentiation. It was reported that* PPAR*γ** Pro12Ala variant was associated with increased insulin sensitivity in the general population and thus may protect an individual from T2D [46]. The* KCNJ11* (potassium inwardly rectifying channel subfamily J, member 11) encodes potassium inwardly rectifier 6.2 subunit (Kir6.2) of the ATP-sensitive potassium (K_ATP_) channel, which has an impact on glucose-dependent insulin secretion in pancreatic *β*-cells [9]. The E23K variant in this gene demonstrated a robust association with T2D using the candidate gene approach [9].* WFS1 *and* HNF1B* were also uncovered as established genes associated with T2D [11, 12].* WFS1* encodes wolframin, a membrane glycoprotein that maintains calcium homeostasis of the endoplasmic reticulum. Rare mutations in* WFS1* cause Wolfram syndrome, which is characterized by a significant *β*-cell loss as a result of enhanced endoplasmic reticulum stress [47–49].* HNF1B* encodes hepatocyte nuclear factor 1 homeobox B, which is a liver-specific factor of the homeobox-containing basic helix-turn-helix family. Mutation of this gene was demonstrated to cause MODY5 [38].

In 2006, a large-scale association study identified* TCF7L2* as an important genetic factor for T2D in Icelandic individuals [10]. This discovery was a significant breakthrough as this association was then widely confirmed in populations of European origin and other ethnic groups, such as Japanese and American individuals [50–57]. Therefore,* TCF7L2 *was regarded as the most significant T2D susceptibility gene identified to date.

#### 2.1.2. Genome-Wide Association Study (GWAS)

With the advent of GWAS, exploration of the genetic basis for T2D susceptibility has made significant breakthroughs. In 2007, the results of five genome-wide association studies were published. These studies increased the number of confirmed T2D susceptibility loci to nine (*PPAR*γ**,* KCNJ11*,* TCF7L2*,* CDKAL1*,* CDKN2A/B*,* IGF2BP2*,* HHEX/IDE*,* FTO,* and* SLC30A8*) [13–18]. Except for* PPAR*γ** and* FTO*, which mainly affect insulin sensitivity, all the other genes may affect *β*-cell function, although the exact mechanisms remain largely unknown [16].* HHEX*, which is located on chromosome 10q, is a member of the homeobox family and encodes a transcription factor that maybe involved in Wnt signaling [58]. Nevertheless, these studies established the utility of GWAS approach in elucidating complex genetic traits.

In 2008, to increase the power of identifying variants with modest effects, a meta-analysis of three GWAS, including Diabetes Genetics Initiative (DGI), Finland-United States Investigation of NIDDM Genetics (FUSION), and Wellcome Trust Case Control Consortium (WTCCC), were conducted. This study detected at least six previously unknown loci that reached genome-wide significance for association with T2D (*P* < 5 × 10^−8^), with the loci being* JAZF1*,* CDC123-CAMK1D*,* TSPAN8-LGR5*,* THADA*,* ADAMTS9*, and* NOTCH2* [19]. Genetic variants in* JAZF1*,* CDC123-CAMK1D*,* TSPAN8-LGR5, and THADA *have been reported to affect pancreatic *β*-cell functions [59, 60].

In 2009, a novel genetic variant rs2943641, which is located adjacent to the insulin receptor substrate 1 gene (*IRS1*), was shown to have a significant association with insulin resistance and hyperinsulinemia and further studies also showed that this variant is implicated in reduced basal* IRS1* protein level and decreased* IRS1*-associated phosphatidylinositol-3-OH kinase activity in human skeletal muscle biopsies [21]. In the same year, a variant near* MTNR1B* was found to be associated with increased fasting plasma glucose level and higher risk of T2D (odds ratio = 1.15, 95% CI = 1.08–1.22, *P* = 6.3 × 10^−5^) [20]. Ten GWAS involving a total of 36,610 individuals of European descent and a meta-analysis of 13 case-control studies replicated this result and found that risk alleles in this gene are associated with reduced *β*-cell function as measured by homeostasis model assessment (HOMA-*β*, *P* = 1.1 × 10^−15^) [61].

In 2010, a meta-analysis of 21 genome-wide association studies performed by Dupuis and colleagues identified* ADCY5*,* PROX1*,* GCK*,* GCKR*, and* DGKB/TMEM195* as new genetic loci for T2D susceptibility [22]. Among these loci,* DGKB/TMEM195*,* GCK*,* PROX1,* and* ADCY5* mainly affect *β*-cell functions, whereas the locus mapped in* GCKR* shows a primary effect on insulin action [22]. In the same year, another genome-wide association study by Qi and colleagues discovered new variants near* RBMS1* and* ITGB6* genes at 2q24, and these variants were found to affect glucose metabolism and insulin resistance [23]. In addition, an expanded meta-analysis of existing GWAS by Voight and colleagues identified 12 new signals with a combined *P* < 5 × 10^−8^, including* BCL11A*,* ZBED3*,* KLF14*,* TP53INP1*,* TLE4*,* CENTD2*,* HMGA2*,* HNF1A*,* PRC1*,* ZFAND6*,* DUSP9, *and* KCNQ1* [24].* HNF1A* was previously recognized as the causal gene of MODY3 [62] and also harbored the common variant (G319S) that contributes to early-onset T2D [63, 64].* DUSP9*, mapped on chromosome X, encodes a member of the family of mitogen-activated protein kinase phosphatase 4, MKP4, which is important in cell cycle regulation and plays pivotal roles in regulating insulin action [65–67].

In 2012, a meta-analysis conducted by Morris and colleagues identified additional ten previously unreported T2D susceptible loci, including* BCAR1*,* MC4R*,* CILP2*,* ANKRD55*,* TLE1*,* KLHDC5*,* MGC21675*,* ANK1*,* ZMIZ1*, and* GRB14* [25]. To assess the potential function of these loci, OGTT was employed to test insulin release and insulin sensitivity.* ANK1* was found to be associated with insulinogenic and disposition indices, indicating that this gene probably had an effect on insulin secretion [68]. In this study,* GRB14 *and* AKNRD55* were associated with decreased Matsuda index, an index of insulin sensitivity [68].

As described above, genetic studies of T2D in European populations have made significant progress in our understanding of T2D susceptibility. However, existing data can only provide partial explanation for the heritability of T2D. It is well known that discrepancies exist in allelic frequencies and effect sizes in different ethnic groups. It is, therefore, important to understand whether these variants are also applicable to other ethnic populations.

### 2.2. Genetics of T2D in East Asians

Epidemiological studies have documented consistent increases in the prevalence of diabetes in Asia, especially in China, with diabetes prevalence having increased from 2.6% in 2000 to 9.7% in 2010 [69]. However, our understanding of the genetic basis of T2D in East Asia remains limited. It is therefore imperative to identify specific genes associated with this disease in East Asians.

In 2008, two papers provided the first reports of GWAS for T2D in East Asian populations and ascertained* KCNQ1* as a new susceptibility locus [70, 71].* KCNQ1* encodes the pore-forming *α*-subunit of the voltage-gated K^+^ channel (KvLQT1), which is expressed mainly in the heart and pancreas. Its association with T2D was further replicated in Korean [72], Chinese [26], and Singaporean [73] populations, as well as individuals of European descent [70]. Therefore,* KCNQ1* is regarded as the most significant locus for T2D in East Asians. This genetic variant is implicated in insulin secretion, which may be the explanation for its association with T2D [73, 74].

In 2010, another GWAS conducted in a Japanese group identified two new loci in* UBE2E2 *and* C2CD4A-C2CD4B*. Genetic variants in* C2CD4A-C2CD4B* were then validated in European populations [27]. When the GWAS reports sprung up in East Asians, Chinese investigators performed their first GWAS in the Han Chinese residing in Taiwan and identified two new susceptible loci for T2D in* PTPRD* (protein tyrosine phosphatase receptor type D) and* SRR* (serine racemase) [29]. PTPRD is a protein tyrosine phosphatase and may play a role in the pathogenesis of T2D through increased insulin resistance [75].* SRR* encodes a serine racemase that synthesizes D-serine from L-serine and which confers risk for T2D via the glutamate signaling pathway [76, 77]. In the same year, a fast-track, multiple-stage study conducted in Han Chinese population by Shu and colleagues discovered a novel genetic susceptibility locus rs1359790, at* 13q31.1* for T2D, and this variant was also validated in European Americans, Koreans, and Singapore Chinese [28].

In 2011, in order to identify additional genes in East Asians, Cho and colleagues carried out a meta-analysis of three-stage GWAS in populations of East Asian descent. Compelling evidence for association with T2D of eight novel loci was demonstrated by this study. All of these loci are mapped in or near* GLIS3*,* PEPD*,* FITM2-R3HDML-HNF4A*,* KCNK16*,* MAEA*,* GCC1-PAX4*,* PSMD6, and ZFAND *[30].

In 2012, another GWAS in Japanese populations revealed that rs515071 in* ANK1* was associated with T2D at the genome-wide significance level [31].* ANK1*, which encodes a member of the ankyrin family, is also reported to be associated with impaired insulin secretion and abnormal level of HbA_1c_ [68, 78]. In addition, GWAS in Beijing and Shanghai populations added two new loci to the list,* GRK5* and* RASGRP1*, and the association signal for* GRK5* seems to be specific to East Asians [32]. GRK5 is regarded as a positive regulator of insulin sensitivity and this protein is a potential therapeutic target for the treatment of insulin resistance [79].

In 2013, a novel variant rs10229583 at 7q32 near* PAX4* was identified in a meta-analysis of three GWAS from Southern Han Chinese descents [33]. As a member of the paired box family of transcription factors,* PAX4 *plays a critical role in pancreatic *β*-cell development and *β*-cell functions [80]. Further three new predisposing loci,* MIR129-LEP*,* GPSM1*, and* SLC16A13*, with genome-wide significance for T2D were identified [34]. Rs791595 is located between* MIR129-1* and* LEP*. The coding product of* LEP*, leptin, is closely related to body weight regulation and its deficiency in mice and human causes morbid obesity and diabetes, while the role of* MIR129* in diabetes remains unknown [81].

Besides these newly identified loci, some susceptible genes identified in Caucasians were also replicated in East Asians, such as* PPAR*γ**,* KCNJ11*, TCF2, TCF7L2,* CDKAL1*,* CDKN2A-CDKN2B*,* IDE-KIF11-HHEX*,* IGF2BP2*, MTNR1B,* SLC30A8*,* KCNQ1*,* CDC123*,* GLIS3*,* HNF1B*,* and DUSP9 *[32, 82–93].

Together, all these T2D risk loci, initially identified or replicated in East Asians, provide new perspectives on the etiology of T2D and uncover the need for further studies to explore additional loci with strong effects on T2D.

### 2.3. Genetics of T2D in South Asians

South Asia, with more than a quarter of the world's population, harbors the highest number of patients suffering from T2D [94]. Currently, the number of diabetic patients is reaching 62.4 million, and the number of prediabetic individuals is reaching 77.2 million [95]. Compared to European populations, South Asians are at a fourfold higher risk of T2D [96, 97]. Therefore, significant efforts should be made to identify common genetic variants underlying the T2D risk in individuals of South Asian ancestry.

In 2011, a GWAS in South Asians identified six novel loci harboring disease-predisposing variants, including* GRB14*,* ST6GAL1*,* VPS26A*,* HMG20A*,* AP3S2,* and* HNF4A*. Single nucleotide polymorphisms (SNPs) at* GRB14* were associated with insulin sensitivity and SNPs at* ST6GAL1* and* HNF4A* were associated with pancreatic *β*-cell function [35].

In 2013, a GWAS performed in Indians identified* TMEM163* on chromosome 2q21 as a new signal for T2D.* TMEM163* encodes a putative vesicular transporter in nerve terminals and shows a plausible effect on T2D by impairing insulin secretion [36]. Concurrently, a novel locus at 13q12 in the* SGCG* gene was identified to confer T2D susceptibility in Punjabi Sikhs from Northern India. This association demonstrated excellent consistency across the three Sikh samples, but no significant association was observed in a large East Asian replication study, indicating that the detected locus is specific to the Indian Punjabi Sikh population [37].

In consideration of India's complex demographic history, cultural diversity, differences in risk allele frequency, and pattern of linkage disequilibrium existing between European and South Asian populations, large replication studies were conducted to evaluate the contribution of European-derived loci in South Asian populations. SNPs in or near* PPARG, KCNJ11, TCF7L2, SLC30A8, HHEX, CDKN2A/B, IGF2BP2, CDKAL1, FTO, KCNQ1, JAZF1, IRS1, KLF14, CHCHD9, *and* DUSP9 *displayed significant associations with T2D in Pakistani populations, with similar effect sizes as those seen in European populations [98–102].

### 2.4. Genetics of Type 2 Diabetes in Other Populations

The discovery of new susceptibility loci for T2D by GWAS in different ethnic groups emphasizes the need to conduct more GWAS based on ethnic background. In addition to European and Asian populations, researchers also conducted studies in Pima Indians and Mexican Americans aimed at identifying new risk loci.

In Pima Indians, a few genes have been reported to confer risk of T2D. In 2007, researchers found that variants within* ARHGEF11 *nominally increased the risk of T2D, possibly as a result of increased insulin resistance [103]. In 2008, variation within* PCLO *was confirmed to have a modest effect on early-onset T2D, possibly by reduction of insulin action [104]. In 2010,* ACAD10 *variation was found to increase T2D risk by impairing insulin sensitivity via abnormal lipid oxidation [105]. Soon afterwards, an* ASK1 *variant was identified to confer susceptibility to T2D by decreasing insulin sensitivity owing to reduced ASK1 expression in skeletal muscle [106]. However, a replication study, which genotyped SNPs mapped in* CDKAL*,* SLC30A8*,* HHEX*,* EXT2*,* IGF2BP2*,* LOC387761*, and* FTO* previously associated with T2D in Caucasians, did not provide any evidence for association with T2D or obesity among full-heritage Pima Indians. Instead, they found that* CDKAL1, HHEX,* and* EXT2* were evidently associated with either insulin secretion or insulin action in Pima Indians with normal glucose tolerance [107].

Similarly, analysis of T2D risk genes in Mexican American populations had identified several novel candidate loci for T2D, such as rs979752 and rs10500641 near* UBQLNL* and* OR52H1* on chromosome 11, rs2773080 and rs3922812 in or near* RALGPS2* on chromosome 1, and rs1509957 near* EGR2* on chromosome 10 [108]. In 2011, the largest GWAS and meta-analysis of T2D in Mexican populations identified 49 SNPs in eight gene regions (*PER3, PARD3B, EPHA4, TOMM7, PTPRD, HNT, LOC729993, and IL34*) and six intergenic regions with an unadjusted *P* value <1 × 10^−5^ [109]. In consideration of the fact that all the above loci did not reach genome-wide significance (*P* < 5 × 10^−8^), Williams and colleagues analyzed 9.2 million SNPs in 8,214 Mexicans and other Latin Americans and identified a novel locus associated with T2D spanning the solute carriers* SLC16A11* (*P* = 3.9 × 10^−13^; odds ratio (OR) = 1.29). They observed that SLC16A11 mainly localizes with the endoplasmic reticulum membrane protein, calnexin, in liver, salivary gland, and thyroid. Importantly, overexpression of SLC16A11 in HeLa cells resulted in substantial increases in triacylglycerol, suggesting that SLC16A11 may have a role in hepatic lipid metabolism [16, 110]. Nevertheless, the role of all these risk loci in the pathogenesis of diabetes remains unclear and needs further investigations.

## 3. Correlation of the Susceptibility Loci with the Pathogenesis of T2D

With the large number of aforementioned genetic loci susceptible to T2D, the question pertains to how they participate in the pathogenesis of T2D. A great number of studies have suggested that genetic variants in or near* KCNJ11, TCF7L2, WFS1, HNF1B, IGF2BP2, CDKN2A-CDKN2B, CDKAL1, SLC30A8, HHEX/IDE, KCNQ1, THADA, TSPAN8/LGR5, CDC123/CAMK1D, JAZF1, MTNR1B, DGKB/TMEM195, GCK, PROX1, ADCY5, SRR, CENTD2, ST6GAL1, HNF4A, KCNK16, FITM2-R3HDML-HNF4A, GLIS3, GRB14, ANK1, BCAR1, RASGRP1, *and* TMEM163 *may confer T2D risk through impaired *β*-cell function [16, 24, 44, 68, 111–114], whereas* PPAR*γ*, ADAMTS9, IRS1, GCKR, RBMS1/ITGB6, PTPRD, DUSP9, HMGA2, KLF14, GRB14, ANKRD55, *and* GRK5* have an impact on insulin action [21, 24, 115, 116] (Tables 1, 2, and 3).* FTO* and* MC4R*, previously identified genes associated with obesity, appear to confer T2D risk through their primary effects on BMI, but recent GWAS have shown that their effects on T2D were independent of BMI, though* FTO* may have a small but detectable influence on T2D risk through insulin action [117, 118].

### 3.1. Impact of* TCF7L2* on the Risk of T2D

*TCF7L2* is the most intensively studied locus for T2D risk so far. The risk alleles of* TCF7L2 *were associated with enhanced expression of this gene in human islets as well as impaired insulin secretion both* in vitro* and* in vivo*. The authors also observed an impaired incretin effect in subjects carrying risk alleles of* TCF7L2* and proposed the engagement of the enteroinsular axis in T2D [119]. Dennis and colleagues then verified this result and indicated that* TCF7L2* variant rs7903146 affected risk of T2D, at least in part, through modifying the effect of incretins on insulin secretion. This was not due to reduced secretion of glucose-dependent insulinotropic polypeptide (GIP) and glucagon-like peptide 1 (GLP-1), which exhibit an important physiological role in boosting insulin secretion following meals, but rather due to the effect of* TCF7L2* on the sensitivity of *β*-cells to incretins [120]. TCF7L2 has also been linked to altered pancreatic islet morphology as exemplified by increased individual islet size and altered alpha and beta cell ratio/distribution within human islets [121]. This phenomenon is also observed in other* in vivo* or* in vitro* studies [122–124]. This further strengthened the evidence for the role of TCF7L2-associated alteration of cell types in islets in the pathogenesis of T2D.

TCF7L2 encodes the transcription factor TCF4 which is related to Wnt signaling pathway and which plays a critical role in the pathogenesis of T2D. The major effector of the canonical Wnt signaling pathway is known as *β*-catenin/TCF. This bipartite transcription factor is formed by free *β*-catenin (*β*-cat) and a member of the TCF protein family, including TCF7L2 (previously known as TCF-4) [125]. GWAS have revealed the involvement of a Wnt ligand (Wnt-5b), Wnt coreceptor (LRP-5), and the Wnt pathway effector TCF7L2 in the development of diabetes [126]. Several previous studies also provide evidence that the *β*-catenin/TCF axis participates in pancreatic cell proliferation and differentiation [127–131]. Treatment of *β*-cells with purified Wnt protein or activated *β*-catenin augmented the proliferation of these cells [132]. Intriguingly, deletion of *β*-catenin within the pancreatic epithelium resulted in an almost complete lack of acinar cells, whereas deletion of *β*-catenin specifically in differentiated acinar cells had no such effect [128], suggesting that the* TCF7L2*-related Wnt signaling mainly perturbs pancreatic growth but not pancreatic function. However, deletion of islet* TCF7L2* expression from *β*-cells did not show any demonstrable effects on glucose-stimulated insulin secretion (GSIS) in adult mice, whereas manipulating* TCF7L2* levels in the liver caused hypoglycemia and reduced hepatic glucose production [133]. In concordance with these results, risk alleles in* TCF7L2* were associated with hepatic but not peripheral insulin resistance and enhanced rate of hepatic glucose production in human [119]. Therefore, TCF7L2-related disruption of *β*-cell function is probably the indirect consequence of primary events in liver or other organs/systems.

### 3.2. Impact of* SCL30A8* on the Risk of T2D

Besides* TCF7L2*, solute carrier family 30 member 8 gene (*SCL30A8*) has also been explored in depth.* SCL30A8 *encodes the islet-specific zinc transporter ZnT-8, which delivers zinc ions from cytoplasm into intracellular insulin-containing granules, and is implicated in insulin maturation and/or storage processes in *β*-cells [134]. Expression level of ZnT-8 was remarkably downregulated in the pancreas of db/db and Akita mice in the early stage of diabetes [135]. Global* SCL30A8 *knockout mice demonstrated reduced plasma insulin, impaired GSIS, and markedly reduced islet zinc content [136]. Remarkably, both ZnT-8 knockout mice and human individuals carrying risk alleles of* SLC30A8* exhibited increased hepatic insulin clearance, with significantly increased c-peptide/insulin ratios [137]. Contrary to the previous findings, overexpression of ZnT-8 in INS-1 cells stimulated zinc accumulation and enhanced GSIS of these cells [138]. Importantly, a recent study discovered that* SCL30A8* gene transcription was regulated by Pdx-1, a *β*-cell-enriched transcription factor, and involved in the development of islets, through an intrinsic enhancer. Restriction of Pdx-1 in pancreatic islet *β*-cells correlated with the induction of* SCL30A8* gene and ZnT-8 protein expression [139]. Therefore, the specific pathways by which* SL30A8* correlates with the pathogenesis of T2D still need further exploration.

It should be noted that a great number of low frequency variants might not be identified by GWAS owing to the required genome-wide significance level. According to the existing studies, many important loci are also obscured as a result of borderline associations. The known variants account for only a small amount of the overall estimated genetic heritability; therefore, there is still a long way to go in terms of understanding the pathogenesis of type 2 diabetes.

## 4. Clinical Utility of Genetic Information: Prediction of Type 2 Diabetes

One of most important clinical utilities of genetic information is to predict the risk of developing T2D among nondiabetic individuals. This will facilitate the early interventional strategies to prevent or delay the onset of the disease. A vast number of recent studies have constructed genetic risk score models by summing up numerous independently inherited susceptible variants for T2D to evaluate the predictive ability from the current genetic information. For example, the area under the receiver operating characteristic (ROC) curves (AUCs) is used to assess discriminative accuracy of this approach. The AUC value can range from 0.5 to 1.0, where the AUC of 0.5 stands for the lack of discrimination and AUC of 1 stands for perfect discrimination. An AUC value of greater than 0.75 is considered to be clinically useful [140]. Imamura and colleagues created a genetic risk score model using 49 susceptibility alleles (GRS-49) for T2D in a Japanese population and discovered an increased level of AUC with combined GRS-49 and clinical factors (including age, sex, and BMI) compared with each individually. But the AUC value is only 0.773, which shows a clinically modest but statistically significant effect on T2D [141]. This phenomenon is also observed in many other studies from different ethnic groups [142, 143]. Controversially, it was proposed that phenotype-based risk models are superior to models based on 20 common independently inherited diabetes risk alleles in discrimination for T2D, with the observation of only minimal improvement in accuracy of risk estimation when adding genotypes to phenotype-based risk models [144]. The discrepancy may result from the fact that prediction for T2D using genetic information is largely affected by age. For example, the Framingham Offspring Study conducted with 3,471 subjects followed over 34 years found out that common genetic variations appropriately reclassified younger people for T2D risk beyond clinical risk factors, but it failed in older people [145]. In addition, along with the rapid economic growth and lifestyle changes, we may underscore the role of environmental factors in the pathogenesis of T2D. A recent study suggested that the potential deleterious effect of several T2D loci may be abolished or at least attenuated by higher physical activity levels or healthy lifestyle, whereas they may be augmented by low physical activity and dietary factors that are similar to a Western dietary pattern [146]. Therefore, these inconsistencies will need further investigations.

## 5. Pharmacogenomics of Type 2 Diabetes

With the advent of GWAS, studies on the roles of inherited and acquired genetic variations in drug response have undergone an evolution from pharmacogenetics into pharmacogenomics, with a shift from the focus on individual candidate genes to GWAS [147]. Clinically, it is often observed that even patients who receive similar antidiabetic regimens demonstrate large variability in drug disposition, glycemic response, tolerability, and incidence of adverse effects [148]. This interindividual variability can be attributed to specific gene polymorphisms involved in the metabolism, transportation, and therapeutic mechanisms of oral antidiabetic drugs. Pharmacogenomics is on the agenda to explore feasible genetic testing to predict treatment outcome, so that appropriate steps could be taken to treat type 2 diabetes more efficiently.

In general, the oral antidiabetic drug (OAD) is the first line treatment for T2D after failure of lifestyle intervention. The most commonly prescribed OADs include sulfonylureas (SU), biguanides, thiazolidinediones (TZDs), glinides, and *α*-glucosidase inhibitors. To date, numerous pharmacogenetic studies comparing these drugs have been conducted in populations with different ethnic backgrounds. With respect to sulfonylureas, genetic variants at multiple loci such as* KCNJ11*,* ABCC8*,* IRIS1*,* TCF7L2*,* NOS1AP*,* KCNQ1*,* CDKAL1*, and* CAPN10* affect pharmacokinetics and/or pharmacodynamics of these drugs [149–157]. Among them,* KCNJ11* encodes a major subunit of the ATP-sensitive K^+^ channel, and* ABCC8* encodes a modulator of ATP-sensitive potassium channels (SUR1). They both play pivotal roles in insulin secretion and are both shown in pharmacogenomic studies to impact sulfonylureas efficacy [151, 158]. The Arg (972) IRS-1 variant is associated with increased risk for secondary failure to sulfonylurea and it is noteworthy that the genotype frequency of this variant is twice as high in patients with secondary failure to sulfonylurea compared to the diabetic patients whose blood glucose levels were well controlled with oral therapy [157]. In diabetic patients carrying risk alleles in* NOS1AP* gene, glibenclamide is less effective in reducing glucose levels. The increased mortality in users of sulfonylurea was also shown in this paper, reminding us of the fact that genetic variation could alter responses to T2D therapy [155]. Consistent with this notion, studies have shown that genetic variants in* SLC22A1, SLC22A2, SLC47A1, SLC47A2, *and* ATM *[159–167] were found to affect metformin efficacy.* SLC22A1* encodes organic cation transporter 1 (OCT1), which participates in the transportation of metformin into hepatocytes.* SLC47A1 *encodes the multidrug and toxin extrusion 1 protein (MATE1), which facilitates metformin excretion from hepatocytes into bile.* ATM*, a gene known to be involved in DNA repair and cell cycle control, plays a role in metformin efficacy upstream of AMPK, and variation in this gene alters glycemic responses to metformin [167].

Gene polymorphisms associated with glinide (repaglinide and nateglinide) responses were mapped in* CYP2C8, SLCO1B1, TCF7L2, CYP3A4, IGF2BP2, SLC30A8, KCNQ1, KCNJ11, NAMPT, UCP2, MDR1, NeuroD1,* and* PAX4 *[168–174]. Among them,* SLCO1B1* is mainly expressed in the basolateral membrane of hepatocytes and can facilitate hepatic uptake of repaglinide [175]; polymorphisms of this gene have significant influence on the pharmacokinetics of repaglinide with reduced pharmacokinetic exposure after a single oral dose administration of 2 mg repaglinide [176]. Thiazolidinediones, also known as glitazones, act as agonists for their molecular target, peroxisome proliferator-activated receptor-*γ* (PPAR-*γ*). The direct antioxidant action of glitazones may contribute to its effect on insulin resistance [177]. Recent studies have also reported several loci involved in the pharmacogenetics of thiazolidinediones, including* PGC-1α*, resistin, adiponectin, leptin, TNF-alpha, and* CYP2C8 *[178–183].

Pharmacogenetic research provides a means to better understand and improve pharmacotherapy. Despite all these advances in the field of pharmacogenetics, adequately designed and rigorously conducted clinical trials are still needed for guiding therapeutic decisions in T2D treatment.

## 6. Conclusion

To date, approximately 70 loci associated with T2D have been identified. Despite this excellent progress, the current knowledge from these genetic data is still not sufficient to support the clinical utility for the prediction, early identification, and prevention of diabetes. As an emerging field, pharmacogenomics aims at exploring possible molecular mechanisms of drugs and specific genetic variants associated with drug efficacy and thus can make contributions for decisions regarding drug selection, dose titration, treatment duration, and avoidance of adverse drug reactions. However, the loci identified so far explain only a small amount of the estimated heritability of type 2 diabetes and the clinical utility of genetic information is still in its preliminary stage. There is no doubt that intensive studies should be conducted to further identify T2D inheritability factors and promote the translation of novel findings from GWAS to clinical application.

## Figures and Tables

**Table 1:** European-derived susceptibility loci for type 2 diabetes.

	Locus	SNP	Chr.	Position	Allele (risk/other)	RAF*	OR	Probable mechanism	
2000	*PPAR*γ** [8]	rs1801282	3	12368125	C/G	0.92	1.14	Insulin action	Candidate and large-scale association study
2003	*KCNJ11* [9]	rs5219	11	17366148	T/C	0.5	1.14	*β*-Cell function	Candidate and large-scale association study
2006	*TCF7L2* [10]	rs7903146	10	114748339	T/C	0.25	1.37	*β*-Cell function	Candidate and large-scale association study
2007	*WFS1* [11]	rs10010131	4	6343816	G/A	0.6	1.11	*β*-Cell function	Candidate and large-scale association study
2007	*HNF1B* [12]	rs4430796	17	rs4430796	A/G	0.47	1.1	*β*-Cell function	Candidate and large-scale association study
2007	*IGF2BP2* [13–15]	rs4402960	3	186994381	T/G	0.29	1.14	*β*-Cell function	GWAS
2007	*CDKN2A-CDKN2B* [13–15]	rs10811661	9	rs10811661	T/C	0.79	1.2	*β*-Cell function	GWAS
2007	*CDKAL1* [13–16]	rs10946398	6	20769013	C/A	0.31	1.12	*β*-Cell function	GWAS
2007	*SLC30A8* [17]	rs13266634	8	118253964	C/T	0.75	1.12	*β*-Cell function	GWAS
2007	*HHEX/IDE* [17]	rs1111875	10	94452862	C/T	0.56	1.13	*β*-Cell function	GWAS
2007	*FTO* [13, 15, 18]	rs8050136	16	rs8050136	A/C	0.45	1.17	Obesity	GWAS
2008	*NOTCH2* [19]	rs10923931	1	120230001	T/G	0.106	1.13	Unknown	GWAS
2008	*ADAMTS9* [19]	rs4607103	3	64686944	C/T	0.761	1.09	Insulin action	GWAS
2008	*THADA* [19]	rs7578597	2	43644474	T/C	0.902	1.15	*β*-Cell function	GWAS
2008	*TSPAN8/LGR5* [19]	rs7961581	12	69949369	C/T	0.269	1.09	*β*-Cell function	GWAS
2008	*CDC123/CAMK1D* [19]	rs12779790	10	12368016	G/A	0.183	1.11	*β*-Cell function	GWAS
2008	*JAZF1* [19]	rs864745	7	28147081	T/C	0.501	1.1	*β*-Cell function	GWAS
2009	*MTNR1B* [20]	rs1387153	11	92313476	T/C	0.283	1.15	*β*-Cell function	GWAS
2009	*IRS1* [21]	rs2943641	2	226801989	C/T	0.633	1.19	Insulin action	GWAS
2010	*DGKB/TMEM195* [22]	rs2191349	7	15030834	T/G	0.333	1.06	*β*-Cell function	GWAS
2010	*GCKR* [22]	rs780094	2	27594741	C/T	0.394	1.06	Insulin action	GWAS
2010	*GCK* [22]	rs4607517	7	44202193	A/G	0.195	1.07	*β*-Cell function	GWAS
2010	*PROX1* [22]	rs340874	1	212225879	C/T	0.492	1.07	*β*-Cell function	GWAS
2010	*ADCY5* [22]	rs11708067	3	124548468	A/G	0.226	1.12	*β*-Cell function	GWAS
2010	*RBMS1/ITGB6* [23]	rs7593730	2	160879700	C/T	0.23	0.9	Insulin action	GWAS
2010	*KCNQ1* [24]	rs231362	11	2648047	G/A	0.52	1.08	*β*-Cell function	GWAS
2010	*DUSP9* [24]	rs5945326	X	152553116	A/G	0.79	1.27	Insulin action	GWAS
2010	*PRC1* [24]	rs8042680	15	89322341	A/C	0.22	1.07	Unknown	GWAS
2010	*ZFAND6* [24]	rs11634397	15	78219277	G/A	0.6	1.06	Unknown	GWAS
2010	*HNF1A* [24]	rs7957197	12	119945069	T/A	0.85	1.07	Unknown	GWAS
2010	*HMGA2* [24]	rs1531343	12	64461161	C/G	0.1	1.1	Insulin action	GWAS
2010	*CENTD2* [24]	rs1552224	11	72110746	A/C	0.88	1.14	*β*-Cell function	GWAS
2010	*CHCHD9* [24]	rs13292136	9	81141948	C/T	0.93	1.11	Unknown	GWAS
2010	*TP53INP1* [24]	rs896854	8	96029687	T/C	0.48	1.06	Unknown	GWAS
2010	*KLF14* [24]	rs972283	7	130117394	G/A	0.55	1.07	Insulin action	GWAS
2010	*ZBED3* [24]	rs4457053	5	76460705	G/A	0.26	1.08	Unknown	GWAS
2010	*BCL11A* [24]	rs243021	2	60438323	A/G	0.46	1.08	Unknown	GWAS
2012	*HMG20A* [25]	rs7177055	15	75,619,817	A/G	0.68	1.08	Unknown	GWAS
2012	*GRB14* [25]	rs13389219	2	165,237,122	C/T	0.6	1.07	Insulin action	GWAS
2012	*ZMIZ1* [25]	rs12571751	10	80,612,637	A/G	0.52	1.08	Unknown	GWAS
2012	*ANK1* [25]	rs516946	8	41,638,405	C/T	0.76	1.09	*β*-cell function	GWAS
2012	*KLHDC5* [25]	rs10842994	12	27,856,417	C/T	0.8	1.1	Unknown	GWAS
2012	*TLE1* [25]	rs2796441	9	83,498,768	G/A	0.57	1.07	Unknown	GWAS
2012	*ANKRD55* [25]	rs459193	5	55,842,508	G/A	0.7	1.08	Insulin action	GWAS
2012	*CILP2* [25]	rs10401969	19	19,268,718	C/T	0.08	1.13	Unknown	GWAS
2012	*MC4R* [25]	rs12970134	18	56,035,730	A/G	0.27	1.08	Unknown	GWAS
2012	*BCAR1* [25]	rs7202877	16	73,804,746	T/G	0.89	1.12	*β*-Cell function	GWAS

*Data were derived from HapMap East Asian or original studies. Position is given for NCBI Build 36.

SNP: single nucleotide polymorphism; Chr.: chromosome; RAF: risk allele frequency; OR: odds ratio.

**Table 2:** Type 2 diabetes susceptibility loci identified in East Asians.

	Locus	SNP	Chr.	Position	Allele (risk/other)	RAF*	OR	Probable mechanism	
2009	*KCNQ1* [26]	rs2237892	11	2796327	C/T	0.683	1.43	*β*-Cell function	GWAS
2010	*UBE2E2* [27]	rs7612463	3	23311454	A/C	0.134	1.19	Unknown	GWAS
2010	*C2CD4A-C2CD4B* [27]	rs7172432	15	60183681	A/G	0.42	1.13	Unknown	GWAS
2010	*SPRY2* [28]	rs1359790	13	79615157	G/A	0.273	1.15	Unknown	GWAS
2010	*CDC123/CAM K1D* [28]	rs10906115	10	12355003	A/G	0.561	1.13	Unknown	GWAS
2010	*SRR* [29]	rs391300	17	2163008	G/A	0.367	1.28	*β*-Cell function	GWAS
2010	*PTPRD* [29]	rs17584499	9	8869118	T/C	0.226	1.57	Insulin action	GWAS
2011	*MAEA* [30]	rs6815464	4	1299901	C/G	0.640	1.13	Unknown	GWAS
2011	*PSMD6* [30]	rs831571	3	64023337	C/T	0.688	1.09	Unknown	GWAS
2011	*ZFAND3* [30]	rs9470794	6	38214822	C/T	0.203	1.12	Unknown	GWAS
2011	*GCC1-PAX4* [30]	rs6467136	7	126952194	G/A	0.182	1.11	Unknown	GWAS
2011	*KCNK16* [30]	rs1535500	6	39392028	T/G	0.398	1.08	*β*-Cell function	GWAS
2011	*PEPD* [30]	rs3786897	19	38584848	A/G	0.547	1.1	Unknown	GWAS
2011	*FITM2-R3HD* [30]	rs6017317	20	42380380	G/T	0.545	1.09	*β*-Cell function	GWAS
2011	*GLIS3* [30]	rs7041847	9	4277466	A/G	0.529	1.1	*β*-Cell function	GWAS
2012	*ANK1* [31]	rs515071	8	41,638,405	C/T	0.8	1.18	Unknown	GWAS
2013	*GRK5* [32]	rs10886471	10	121139393	C/T	0.756	1.12	Insulin action	GWAS
2013	*RASGRP1* [32]	rs7403531	15	36610197	T/C	0.317	1.1	*β*-Cell function	GWAS
2013	*PAX4* [33]	rs10229583	7	127034139	G/A	0.829	1.18	Unknown	GWAS
2013	*MIR129-LEP* [34]	rs791595	7	127650038	A/G	0.08	1.17	Unknown	GWAS
2013	*SLC16A13* [34]	rs312457	17	6881117	G/A	0.078	1.2	Unknown	GWAS
2013	*GPSM1* [34]	rs11787792	9	138371969	A/G	0.874	1.15	Unknown	GWAS

*Data were derived from HapMap East Asian or original studies. Position is given for NCBI Build 36.

SNP: single nucleotide polymorphism; Chr.: chromosome; RAF: risk allele frequency; OR: odds ratio.

**Table 3:** Type 2 diabetes susceptibility loci identified in South Asians.

	Locus	SNP	Chr.	Position	Allele (risk/other)	RAF*	OR	Probable mechanism	
2011	*ST6GAL1* [35]	rs16861329	3	188149155	G/A	0.86	1.09	*β*-Cell function	GWAS
2011	*HNF4A* [35]	rs4812829	20	42422681	A/G	0.29	1.09	*β*-Cell function	GWAS
2011	*VPS26A* [35]	rs1802295	10	70601480	A/G	0.26	1.08	Unknown	GWAS
2011	*AP3S2* [35]	rs2028299	15	88175261	C/A	0.31	1.1	Unknown	GWAS
2011	*HMG20A* [35]	rs7178572	15	75534245	G/A	0.52	1.09	Unknown	GWAS
2011	*GRB14* [35]	rs3923113	2	165210095	A/C	0.74	1.09	Insulin action	GWAS
2013	*TMEM163* [36]	rs998451	2	135145758	G/A	1	1.56	*β*-Cell function	GWAS
2013	*SGCG* [37]	rs9552911	13	22762657	A/G	0.07	0.67	Unknown	GWAS

*Data were derived from HapMap East Asian or original studies. Position is given for NCBI Build 36.

SNP: single nucleotide polymorphism; Chr.: chromosome; RAF: risk allele frequency; OR: odds ratio.
